# Exploring the Effects of the Interaction of Carbon and MoS_2_ Catalyst on CO_2_ Hydrogenation to Methanol

**DOI:** 10.3390/ijms23095220

**Published:** 2022-05-07

**Authors:** Pingping Cui, Ruyu Sun, Linfei Xiao, Wei Wu

**Affiliations:** National Center for International Research on Catalytic Technology, Key Laboratory of Chemical Engineering Process & Technology for High-Efficiency Conversion, College of Heilongjiang Province, School of Chemistry and Material Sciences, Heilongjiang University, Harbin 150080, China; c2191131pp@163.com (P.C.); sry1999sun@163.com (R.S.)

**Keywords:** MoS_2_@C, carbon dioxide, hydrogenation, methanol

## Abstract

Hydrogenation of CO_2_ to form methanol utilizing green hydrogen is a promising route to realizing carbon neutrality. However, the development of catalyst with high activity and selectivity to methanol from the CO_2_ hydrogenation is still a challenge due to the chemical inertness of CO_2_ and its characteristics of multi-path conversion. Herein, a series of highly active carbon-confining molybdenum sulfide (MoS_2_@C) catalysts were prepared by the in-situ pyrolysis method. In comparison with the bulk MoS_2_ and MoS_2_/C, the stronger interaction between MoS_2_ and the carbon layer was clearly generated. Under the optimized reaction conditions, MoS_2_@C showed better catalytic performance and long-term stability. The MoS_2_@C catalyst could sustain around 32.4% conversion of CO_2_ with 94.8% selectivity of MeOH for at least 150 h.

## 1. Introduction

Carbon dioxide is one of the important greenhouse gases contributing to global warming, glacial melting, sea-level rise, ocean acidification, and hypercapnia [1,2]. While CO_2_ is also an inexpensive, abundant, sustainable, and renewable C1 resource [3,4], it can be captured and utilized in a rational way to form high value-added chemicals, such as carbonate, methanol, formic acid, olefins, aromatics, and so on [5,6]. CO_2_ conversion to MeOH is the most direct route for synthesizing oxygenated compounds and has received great interest [7,8,9]. As a feedstock, methanol can be used as a precursor for synthesizing aromatics and low olefins; moreover, it is also considered to be a green hydrogen carrier and is used as a fuel additive and fuel substitute directly [10]. Although the synthesis of MeOH from CO_2_ and H_2_ is exothermic, CO_2_ conversion to MeOH is kinetically limited at low temperatures and thermodynamically limited at high temperatures. Due to a high activation energy barrier for the cleavage of the C-O bonds in CO_2_, which clearly results in a chief challenge to developing effective catalysts for the synthesis of methanol from CO_2_ at a low temperature.

Until now, there are numerous endeavors on different catalyst systems to address CO_2_ hydrogenation to methanol, such as Cu-based catalysts [11,12,13], precious metal catalysts [14,15,16,17,18], In_2_O_3_-based catalysts [19,20,21], solid solution catalysts [22,23,24], and so on. Among these catalysts, the Cu/ZnO/Al_2_O_3_ catalyst has been used as an industrial catalyst for methanol synthesis from CO_2_ hydrogenation. Therefore, Cu-based catalysts have been extensively investigated in CO_2_ hydrogenation to methanol and the Cu-ZnO composite is employed as the active species in more than 60% of related reports [25]. However, Cu-based catalysts showed lower selectivity and poor stability because of the competing reverse water-gas shift reaction and the sintering of the active phase, and it was exacerbated by the hydrophilicity of Al_2_O_3_, which could adsorb water generated from the CO_2_ hydrogenation. Therefore, there was an urgent need to develop highly efficient catalysts for CO_2_ hydrogenation to methanol.

As a typical two-dimensional lamellar material, MoS_2_ shows magic physical and chemical properties and is consequently applied in catalysts for hydrogen evolution reactions in electrocatalysis, hydrodesulfurization, and synthesis gas conversions. Early in 1981, Saito and Anderson used MoS_2_ as a catalyst for CO_2_ hydrogenation at 350 °C, 1 atm, and H_2_/CO_2_ = 3.74 [26], while CO was the sole product due to the water gas shift reaction. Combining the electrical conductivity of graphenes with the catalytic activity of MoS_2_, a few layers of MoS_2_ platelets supported on few-layers graphene exhibited high catalytic activity for CO_2_ hydrogenation, but the major product was methane, frequently with selectivity above 95% and in some cases close to 100% [27]. The catalytic performance of MoS_2_ for CO_2_ hydrogenation has been studied by density functional theory (DFT) and calculations suggest that MoS_2_ could promote the C-O scission of HxCO intermediates, thus explaining the high selectivity of hydrocarbons in the CO_2_ hydrogenation process by using molybdenum sulfides as a catalyst [28]. Interestingly, MoS_2_ has been used as a support for a single atom in the hydrogenation of CO_2_. The main product was methanol [29]. Zeng et al. have reported that the MoS_2_ supporting isolated Pt monomers favored the conversion of CO_2_ into methanol, and the selectivity of methanol arrived at 95.4% [30]. These results were thanks to the synergetic interaction between neighboring Pt monomers on MoS_2_. Recently, Wang et al. [31] found that the sulfur vacancy played a key role in the adsorption and activation of CO_2_ when the sulfur vacancy-rich MoS_2_ was used as a catalyst for the hydrogenation of CO_2_. At 180 °C, the selectivity of methanol was achieved at 94.3% with a 12.5% CO_2_ conversion at 3000 mL g_cat._^−1^ h^−1^ and the catalyst exhibited high stability over 3000 h without any deactivation. However, improving the catalytic performance of molybdenum sulfide in CO_2_ hydrogenation to form methanol is still a challenging topic.

Due to anisotropy, the average slab length and layer stacking were important for describing any catalytic active edge sites of MoS_2_. Therefore, adjusting the slab length and layer stacking of MoS_2_ would be an effective strategy to generate more active edge sites. Abundant strategies were designed to develop nano-scaled MoS_2_ with highly exposed active edge sites to enhance its catalytic activity. In our previous work, MoCS@NSC has been prepared and showed the 97.3% selectivity of MeOH and a 20.0% conversion of CO_2_ in the CO_2_ hydrogenation [32]. In this catalyst, nano-sized MoS_2_ was in-situ generated in the process of preparing nano-sized Mo_2_C confined in carbon material by the pyrolysis of ionic liquid precursors, but its effects on the CO_2_ hydrogenation to form methanol were not clear. In the present work, carbon-confining molybdenum sulfide (MoS_2_@C) was designed and prepared using glucose as a carbon source by the in-situ pyrolysis method. On the one side, the carbon layer coating the surface of MoS_2_ could improve the adsorption quantity of CO_2_; on the other side, MoS_2_ with few layers and little size was prepared by the confinement effect of carbon, which exposed the more active edge sites. As a result, the catalytic performance of MoS_2_ in CO_2_ hydrogenation to form methanol would be improved. Moreover, the influence of the interaction between MoS_2_ and the carbon coating layer on the CO_2_ hydrogenation was investigated, and the activation and the conversion route of CO_2_ in the presence of MoS_2_@C were also discussed by in situ diffuse reflectance infrared Fourier transform spectroscopy (DRIFTS).

## 2. Experiment

### 2.1. Materials

Glucose, ammonium molybdate, and thiourea (NH_4_)_6_Mo_7_O_24_·4H_2_O, AMT were received from Tianjin Kemiou Chemical Reagent Co., Ltd. (Tianjin, China). CO_2_ (99.99%) and 5% Ar/95% H_2_ (99.99%) were obtained from Qing Hua Gas Company Limited (Harbin, China), and all reagents were unused and not further purified.

### 2.2. Catalyst Preparation

#### 2.2.1. Synthesis of MoS_2_

The MoS_2_ was synthesized according to the literature [31] and as follows: ammonium molybdate (1235.9 mg) and thiourea (2283.6 mg) were dissolved in 20 mL of distilled water. Then the resulting solution was placed in the glass evaporating dish at 80 °C for 12 h, and the dried mixture was calcined at 550 °C for 2 h in a nitrogen atmosphere, thus generating MoS_2_.

#### 2.2.2. Synthesis of MoS_2_@C

Ammonium molybdate (1235.9 mg), thiourea (2283.6 mg), and glucose (9458.4 mg) (the molar ratio of carbon to molybdenum was 45) were dissolved in 35 mL of distilled water. Then the resulting solution was placed in the glass evaporating dish at 80 °C for 12 h, and the dried mixture was calcined at 550 °C for 2 h in a nitrogen atmosphere. Finally, the MoS_2_-45@C was obtained.

According to the same processes, the MoS_2_-5@C sample was prepared when the molar ratio of carbon to molybdenum was reduced to 5 in the precursor.

#### 2.2.3. Synthesis of MoS_2_/C

MoS_2_/C catalyst was prepared by the isovolumic impregnation method. Ammonium molybdate (1235.9 mg) and thiourea (2283.6 mg) were dissolved in distilled water. The coconut shell charcoal was added to the solution to achieve a 5% wt. loading. Then the solid was dried at 80 °C and calcined at 550 °C for 2 h under a nitrogen atmosphere. After this process, the MoS_2_/C was prepared.

### 2.3. Catalyst Characterization

The crystalline phase of the catalyst was characterized by X-ray diffraction (XRD) on a D8 Advance with an acceleration voltage of 40 kV.

The microstructure of the catalyst was observed by transmission electron microscopy (TEM) on a JEM-2100 with an acceleration voltage of 200 kV.

The electronic properties of the catalyst surface were determined by X-ray photoelectron spectroscopy (XPS) with an ESCALAB 25, monochromatic Al Kα-rays as the X-ray source, and energy of 1486.6 eV.

The CO_2_-programmed temperature desorption (TPD) was performed. A 0.2 g sample was purged at 500 °C for 60 min under He (40 mL/min). It was naturally cooled to 50 °C and adsorbed for 60 min under CO_2_ (40 mL/min). The sample was then purged for 30 min in He (40 mL/min) and finally warmed from 50 °C to 400 °C at 10 °C/min in He (40 mL/min) for CO_2_ desorption.

In-situ diffuse reflectance and infrared Fourier transform spectroscopy (DRIFTS) measurements were carried out on a Frontier spectrometer by PerkinElmer. The sample was placed directly in the in-situ cell with a ZnSe window and pretreated at 400 °C for 60 min with an H_2_ flow of 20 mL/min, and then the background spectrum of the sample was collected from 500 to 4000 cm^−1^. The feed gas H_2_/CO_2_ (3/l, 60 mL/min H_2_, 20 mL/min CO_2_) was introduced into the cell. The in-situ DRIFTS were recorded with a resolution of 4 cm^−1^ and with an accumulation of 32 scans every 1 min.

### 2.4. Catalytic Performance Test

The activity measurements for CO_2_ hydrogenation were performed in a continuous flow high pressure fixed bed reactor (12 mm internal diameter). Prior to the reaction, the catalyst was pretreated in situ for 3 h at 400 °C in pure hydrogen (22 mL/min). After the reactor had cooled to 220 °C, feed gas with an H_2_/CO_2_ ratio of 3/l and a pressure of 3.0 MPa was introduced into the reactor. The effluent was quantified using a Tianmei GC-7900 F and a GC-7890-II gas chromatograph equipped with a flame ionization detector and a thermal conductivity detector, respectively.

### 2.5. Calculation of CO_2_ Conversion and Product Selectivity

The CO_2_ conversion was calculated by an internal normalization method, and the following Equations (1)–(5) were used for calculating CO_2_ conversion and product selectivity.

The CO_2_ conversion is expressed as $\text{Conv}._{{\text{CO}}_{2}}$ and the selectivity of the products CO, CH_4_, CH_3_OH and CH_3_OCH_3_ is expressed as $\mathrm{Sel}._{\mathrm{CO}},\mathrm{Sel}._{{\mathrm{CH}}_{4}}$, $\mathrm{Sel}._{{\mathrm{CH}}_{3}\mathrm{OH}}\;\mathrm{and}\;\mathrm{Sel}._{{\mathrm{CH}}_{3}{\mathrm{OCH}}_{3}}$ respectively (Equations (1)–(5)). $A_{{\mathrm{CO}}_{2,}\mathrm{out}},A_{\mathrm{Ar},\mathrm{in}},A_{\mathrm{Ar},\mathrm{out}}$ are the peak areas of the CO_2_ and Ar signals at the inlet and tail, in the following order. $f_{{\mathrm{CO}}_{2}}$ and $f_{\mathrm{Ar}}$ are the correction factors for CO_2_ and Ar, in the order of precedence. $A_{CO_{,}out}\;$ and $f_{CO}$, in turn, are the tailpipe signal response area and correction factor for CO. $n_{CH_{4,}out},n_{CH_{3}OH,out},n_{CH_{3}OCH_{3},out}$ in order, represent the molarity at the tail gas of CH_4_, CH_3_OH, and CH_3_OCH_3_. $\;n_{CO_{2,}in}\;and\;n_{CO_{2,}out}$ are the order of the moles of CO_2_ inlet and tail gas.

(1) $$Conv._{CO_{2}}=\frac{\frac{A_{CO_{2,}in}f_{CO_{2}}}{A_{Ar,in}f_{Ar}}-\frac{A_{CO_{2,}out}f_{CO_{2}}}{A_{Ar,out}f_{Ar}}}{\frac{A_{CO_{2,}in}f_{CO_{2}}}{A_{Ar,in}f_{Ar}}}\times 100\%\;$$

(2) $$Sel._{CO}=\frac{\frac{A_{CO_{,}out}f_{CO}}{A_{Ar,out}f_{Ar}}}{\frac{A_{CO_{2,}in}f_{CO_{2}}}{A_{Ar,in}f_{Ar}}-\frac{A_{CO_{2,}out}f_{CO_{2}}}{A_{Ar,out}f_{Ar}}}\times 100\%$$

(3) $$Sel._{CH_{4}}=100\%\times n_{CH_{4,}out}/\left(n_{CO_{2,}in}-n_{CO_{2,}out}\right)$$

(4) $$Sel._{CH_{3}OH}=100\%\times n_{CH_{3}OH,out}/\left(n_{CO_{2,}in}-n_{CO_{2,}out}\right)\;$$

(5) $$Sel._{CH_{3}OCH_{3}}=100\%\times 2n_{CH_{3}OCH_{3},out}/\left(n_{CO_{2,}in}-n_{CO_{2,}out}\right)$$

In addition, the space time yield (STY) of CH_3_OH was calculated according to the following Equation (6):

(6) $$STY=\frac{FMY}{V_{m}W}$$

where F is the volumetric flow rates of CO_2_,  M  is the molecular mass of CH_3_OH, $V_{m}$ is the molar volume of an ideal gas at standard temperature and pressure (22.414 L/mol), W is the mass of catalyst, and Y is the yield of CH_3_OH, respectively.

## 3. Results and Discussions

**Figure 1:**
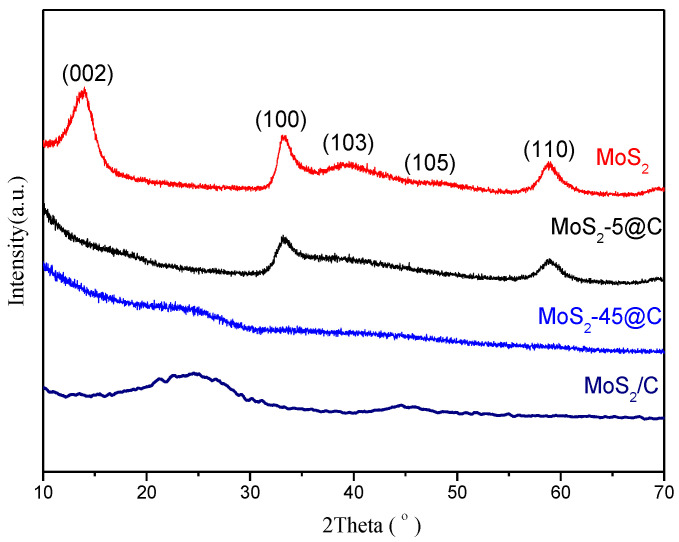
XRD patterns of MoS_2_ samples.

The crystal phase structure of MoS_2_ samples was confirmed by the XRD patterns and is shown in Figure 1. The XRD characteristic diffraction peaks of the bulk MoS_2_ were shown at 14.4°, 32.9°, 39.5°, 49.8°, 58.8°, and 69.2°, which were assigned to (002), (100), (103), (105), (110), and (108) crystalline planes of MoS_2_ [33]. When MoS_2_ was supported on the coconut shell charcoal, except for the characteristic diffraction peak at 2θ of 26° attributed to graphitic carbon [34], there was also a weaker diffraction peak at 2θ of 44.2°, which was assigned to the (105) crystallographic plane of MoS_2_. It was suggested that MoS_2_ was successfully supported on coconut shell charcoal. When the molar ratio of carbon to molybdenum is 45 in the precursor, the resulting MoS_2_-45@C only shows a broad diffraction peak at 2θ of 26°, which corresponds to the (002) planar diffraction peak of graphite [34]. While the characteristic diffraction peaks of MoS_2_ were not apparent, it was due to the high dispersion of MoS_2_.

When the molar ratio of carbon to molybdenum was decreased to 5 in the precursor, the characteristic diffraction peaks of MoS_2_-5@C shown at 32.9°, 39.5°, and 58.8° were attributed to the (100), (103), and (110) crystallographic planes of MoS_2_, respectively. These results confirmed that the carbon, which was formed by in-situ pyrolysis, showed the confinement effect on the synthesis of MoS_2_.

It also can be seen that all patterns are characterized by broad reflections with low intensities, and these observations are clear hints for the poor crystallinity and sizes of coherent scattering domains within the nano regime. Compared with the bulk MoS_2_ sample, the intensity of the (002) reflection at 2θ of 14° disappeared for the samples MoS_2_/C, MoS_2_-5@C, and MoS_2_-45@C. This observation can be explained by the decreasing number of stacked MoS_2_ slabs in the products [35]. These results also indicated the confinement effects of in-situ formation carbon could suppress the growth of MoS_2_ grains.

**Figure 2:**
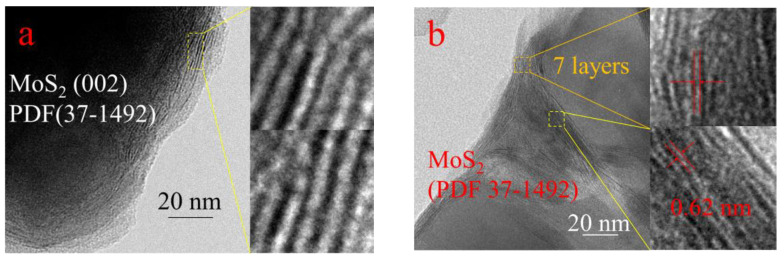

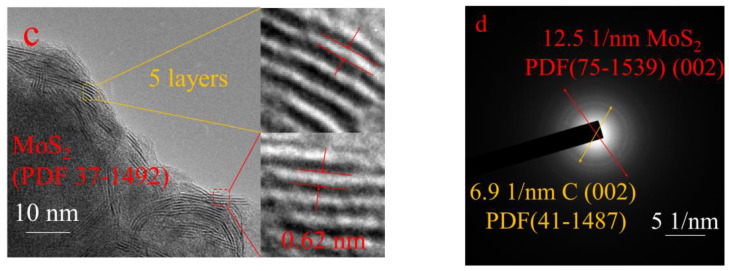
Catalyst characterization: (**a**) MoS_2_ TEM (20 nm); (**b**) MoS_2_/C TEM (20 nm); (**c**) MoS_2_-45@C HRTEM (10 nm); (**d**) MoS_2_-45@C SAED (5 l/nm).

To further enlighten the structure of MoS_2_, transmission electron microscopy (TEM) and high-resolution TEM (HRTEM) analyses were also carried out. From Figure 2a, it can be seen that the bulk MoS_2_ has more layers on the edge, and the thicker part in the middle is not even visible. The clear lattice striations on the surface clearly show that the crystal plane spacing is 0.62 nm, corresponding to the (002) crystal plane of MoS_2_. Compared with bulk MoS_2_, MoS_2_/C is limited by the pores of the coconut shell charcoal (Figure 2b), and the layer number of MoS_2_ in MoS_2_/C was decreased to 7, which corresponded to the results of XRD. These results suggested that loaded on the support was beneficial to reduce the layers of MoS_2_ [36,37,38]. The image of Figure 2c showed MoS_2_ with fewer layers and the smaller practical was obtained successfully in MoS_2_-45@C when the carbon was in situ formed from the pyrolysis of glucose, and it was beneficial to expose the more active site at the edges for the CO_2_ hydrogenation. The SAED patterns (Figure 2d) demonstrate the low crystallinity of MoS_2_, which matches the XRD results.

An X-ray photoelectron spectroscopy (XPS) characterization was carried out to further study the element chemical states in MoS_2_ samples. Figure S1 and Table S1 showed the XPS survey spectra of the samples under study, and the numerical values of the surface composition obtained from these spectra were given in Table S1. The elements of Mo, S, C, and O were detected on the surface of all samples, and the N element was also found on the surface of MoS_2_/C and MoS_2_-45@C. Note that the ratio of the Mo and S in bulk MoS_2_ followed the chemical formula, while the ratio of the Mo and S in MoS_2_/C and MoS_2_-45@C was lower than the chemical formula. These results indicate that the carbon material was doped by the S element when the MoS_2_ was generated in MoS_2_/C and MoS_2_-45@C.

**Figure 3:**
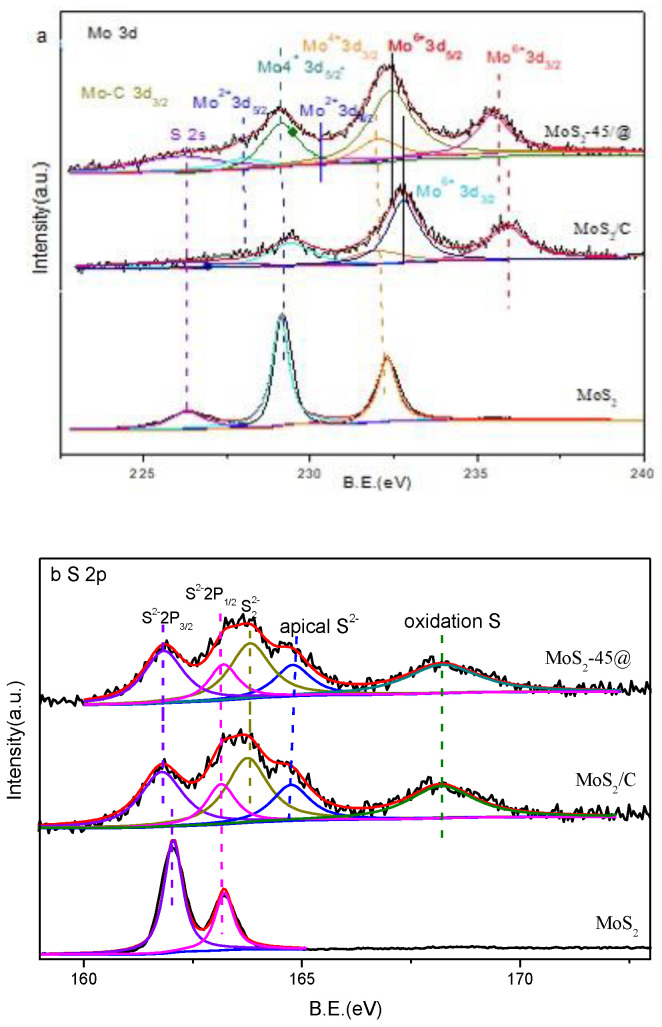
XPS of different catalyst: (**a**) Mo3d and (**b**) S2p.

Figure 3a and Table S2 show the high-resolution XPS spectrum in the Mo 3d region, and the Mo 3d peaks of the bulk MoS_2_ sample with binding energies of 232.0 and 229.1 eV are indexed to Mo 3d_3/2_ and Mo 3d_5/2_, respectively, indicating the presence of Mo^4+^ of molybdenum disulfide [39]. In addition, a small peak at 226.1 eV was found and assigned to S 2s [39]. In XPS of MoS_2_/C, there are two prominent peaks assigned to Mo 3d_3/2_ and Mo 3d_5/2_ (232.0 eV and 229.1 eV), which demonstrate the existence of Mo^4+^ and the successful synthesis of MoS_2_ [40]. Additionally, the peaks at 232.8 and 235.9 eV can be ascribed to Mo^6+^ 3d_5/2_ and 3d_3/2_, which were formed by the surface oxidation of MoS_2_ [41,42,43]. Compared with MoS_2_/C, the characteristic peaks corresponding to Mo^4+^ and Mo^6+^ were also found, but the binding energy between the Mo^6+^ 3d_5/2_ and 3d_3/2_ in MoS_2_-45@C gave a negative shift (0.4 eV), suggesting that the electron interactions between carbon and the MoS_2_ surface in MoS_2_-45@C were stronger than in MoS_2_/C. It is worth mentioning that the two obvious peaks centered at 231.1 and 228.0 eV imply the existence of a C-Mo bond, further confirming the stronger interfacial interaction between the MoS_2_ surface and the carbon coating layer in MoS_2_-45@C [44], which could weaken the Mo-S bond [45].

As exhibited in Figure 3b and Table S3, the S 2p spectra of the bulk MoS_2_ sample showed two strong peaks at 163.2 eV and 161.8 eV for the S2p_1/2_ and S2p_3/2_ binding energies of S^2−^ [46,47]. The electron binding energy of S 2p_3/2_ of S^2−^ in MoS_2_/C and MoS_2_-45@C had a negative shift of about 0.3 eV compared with MoS_2_, respectively. It indicated the existence of electron interactions between the carbon coating layer and the MoS_2_ surface. Moreover, the doublet peaks at 163.7 and 164.8 eV were found, and they were assigned to S_2_^2−^ and apical S^2−^, which indicates the formation of sulfur vacancies on the catalyst surface [48]. The sulfur vacancies could induce the charge-density redistribution, thus producing much more active sites on the catalyst surface. Except, so far, the peak with a binding energy of 168.3 eV was detected in the XPS of MoS_2_/C and MoS_2_-45@C, which resulted from the surface oxidation of sulfur elements, and it was contributed to the presence of a sulfate group [49].

In addition, three peaks were present at 395.1 eV, 398.2–398.6 eV, and 400.1–400.5 eV in the N1s high-resolution XPS spectrum of MoS_2_-45@C and MoS_2_/C (Figure S2 and Table S4), which can be assigned to Mo3p, pyridinic-N, and pyrrolic-N [49]. It was mentioned that the binding energy of pyridinic-N and pyrrolic-N in MoS_2_-45@C was 0.4 eV lower than that in MoS_2_/C, demonstrating an increased electron cloud density around nitrogen in MoS_2_-45@C, which was benefited by the adsorption of CO_2_ on its surface.

Moreover, observing the C 1s spectra of MoS_2_-45@C, it was clearly divided into five fitted peaks at 284.5, 285.2, 286.1, 286.6, and 288.6 eV, which can be related to C-C, C-O, C-O-C/S, C-O-Mo, and C=O (Figure S3 and Table S5). It shows that MoS_2_ could also be tightly linked with carbon by the C-O-Mo bond, being conducive to the MoS_2_ confined in the carbon coating layer [44]. As shown in Figure S4 and Table S6, the peaks in the O1s spectrum at 533.3 eV, 532.5 eV, 531.7 eV, and 530.8 eV are for the Mo-O, C-OH, C-O/O-C-N, and O=C groups, respectively [50].

**Figure 4:**
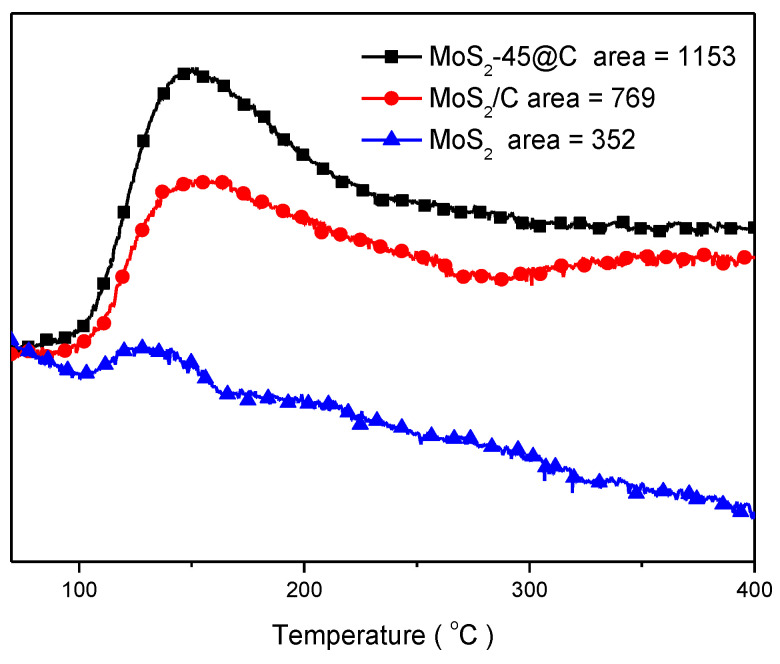
CO_2_-TPD curves of MoS_2_-45@C, MoS_2_/C and MoS_2_.

Due to the dual-site mechanism for CO_2_ hydrogenation, the adsorption and the activation of CO_2_ occur on the surface of the supporter, implying that the activity and conversion of CO_2_ are closely related to the surface basicity of the supporter [25,51,52,53,54]. The CO_2_-TPD experiments were carried out, and the results are shown in Figure 4 and Figure S5. The temperature of the CO_2_ desorption peak was increased with the order bulk MoS_2_ < MoS_2_/C < MoS_2_@C < NSC (N,S-codoping carbon), and the area of the CO_2_ desorption peak was also raised in the same order, suggesting the basic strength and the number of basic sites were enhanced with the order bulk MoS_2_ < MoS_2_/C < MoS_2_@C < NSC. It was due to the strong acid-base interactions between the basic S-C functional group and the acidic CO_2_ molecule in the carbon skeleton and the strong dipole-dipole interaction between the large quadrupole moment of the CO_2_ molecule and the polar sites associated with the sulfur functional group [55].

Under 220 °C, 3 MPa, and a gas hourly space velocity (GHSV) of 5670 mL h^−1^ g_cat._^−1^, the catalytic performance was evaluated in a fixed-bed reactor, and the results are listed in Table 1. As a reference catalyst, the catalytic performance of NSC was investigated first, and the result showed CO_2_ could not be converted, suggesting that NSC had no catalytic activity in the CO_2_ hydrogenation, although it exhibited the highest CO_2_ adsorption capacity. Employing MoS_2_ as a catalyst, the selectivity of methanol arrived at 66.9% with the 18.3% conversion of CO_2_, and methane, as the major byproduct, was found with a 32.7% selectivity. Meanwhile, dimethyl ether (DME) was also detected with a 0.4% selectivity. These results indicated that MoS_2_ displayed catalytic activity for CO_2_ hydrogenation, but the selectivity of methanol was lower, and the high selectivity of methane was given. Using MoS_2_-45@C as an alternative catalyst, the selectivity of methanol was improved to 95.8% with the 27.3% conversion of CO_2_, the selectivity of methane was reduced to 4.2%, and DME was not detected. Additionally, the STY of methanol arrived at 0.538 gg_cat._^−1^ h^−1^ in the presence of MoS_2_-45@C. On the one hand, exposing the more active sites to thin and small MoS_2_ and the higher carbon dioxide adsorption capacity of MoS_2_-45@C were beneficial for accelerating the CO_2_ conversion due to the dual-site mechanism for CO_2_ hydrogenation, and the complete decomposition of CO_2_ on the surface of MoS_2_-45@C was inhibited by the higher carbon dioxide adsorption capacity, which was conducive to improving methanol selectivity [56,57,58]. On the other hand, the stronger interaction of MoS_2_ and the carbon coating layer in MoS_2_-45@C was also beneficial to CO_2_ hydrogenation by decreasing the Gibbs free energy of hydrogen adsorption [43]. Moreover, the high selectivity of methanol was also thanks to the additional S-vacancy in the MoS_2_-45@C catalyst (Figure S6) [31]. When the molar ratio of carbon to molybdenum was decreased to 5 in the precursor, the MoS_2_-5@C gave a 79.9% selectivity of methanol with an 18.4% conversion of CO_2_. These results were probably because the larger size of MoS_2_ and the less active site were exposed. The compared catalyst, MoS_2_/C, showed a 78.5% selectivity of methanol, while the conversion was very low (only 4.2%). This was due to the fact that the 5% loading capacity of MoS_2_ on the coconut shell carbon was too low, which resulted in a large amount of adsorbed CO_2_ that could not be efficiently converted.

**Figure 5:**
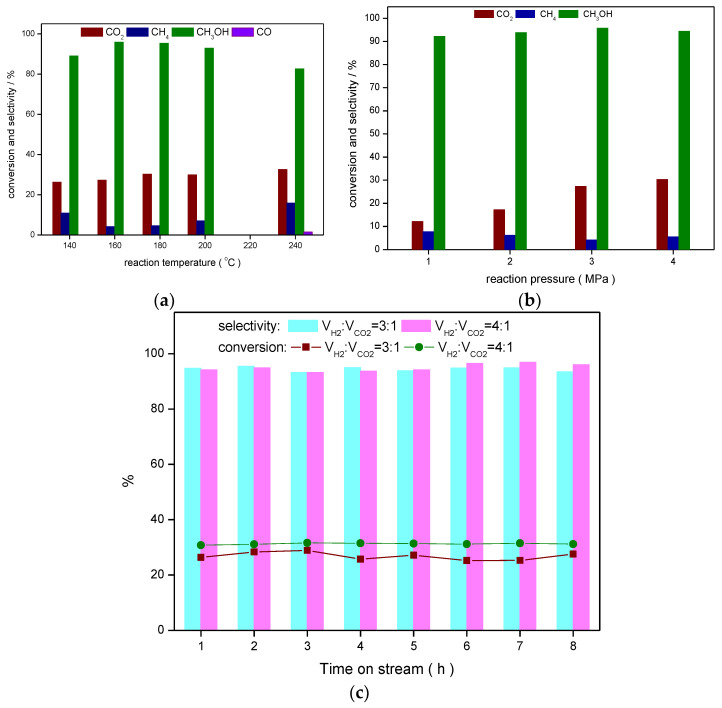
Optimized reaction conditions: (**a**) reaction temperatures, (**b**) reaction pressures, (**c**) ratio of H_2_ to CO_2_.

In the presence of MoS_2_-45@C, the reaction conditions were optimized, and the results are shown in Figure 5. Firstly, the effects of reaction temperature on the catalytic performance were investigated (Figure 5a).

It can be seen that the CO_2_ conversion was increased by enhancing the reaction temperature, and it was improved from 26.3% to 32.6% by raising the temperature from 140 °C to 240 °C. These results indicated that MoS_2_-45@C exhibited higher catalytic activity at a low temperature. When the reaction was performed at 140 °C, 89.0% selectivity of methanol was given, and the methane selectivity reached 11.0%. With increasing reaction temperature to 160 °C, the selectivity of methanol was improved to 95.8%. Further raising the reaction temperature to 240 °C, the selectivity of methanol was reduced, and an 82.6% selectivity of methanol was obtained. It was probably because the too high reaction temperature contributed to the full decomposition of CO_2_ on the catalyst surface and enhanced the catalytic hydrogenation activity, which contributed to the high selectivity of methane. Additively, CO was found when the reaction was performed at 240 °C in the presence of MoS_2_-45@C.

Controlling the reaction temperature at 160 °C, the effects of pressure on the CO_2_ hydrogenation were evaluated by using MoS_2_-45@C as a catalyst (Figure 5b). With increasing the reaction pressure, the CO_2_ conversion was gradually increased with no significant change in methanol selectivity. These results suggest the higher reaction pressure has a positive effect on the CO_2_ conversion. It was because the CO_2_ hydrogenation was a volume reduction reaction. At the same time, the higher reaction pressure was advantageous for the adsorption of CO_2_ on the surface of MoS_2_-45@C, which was favorable for the conversion of CO_2_. In Figure 5b, it is worth noting that the 12.2% conversion of CO_2_ and the 92.2% selectivity of methanol were given when the reaction pressure was reduced to 1 MPa. These results suggested that MoS_2_-45@C displayed a highly catalytic performance under the low reaction pressure. Following that, the influence of the ratio of H_2_ and CO_2_ in the feed gas on the CO_2_ hydrogenation was also investigated (Figure 5c). The results showed the CO_2_ conversion was susceptible to the ratio of H_2_ and CO_2_ in the feed gas, while the selectivity of methanol was kept almost unchanged.

**Figure 6:**
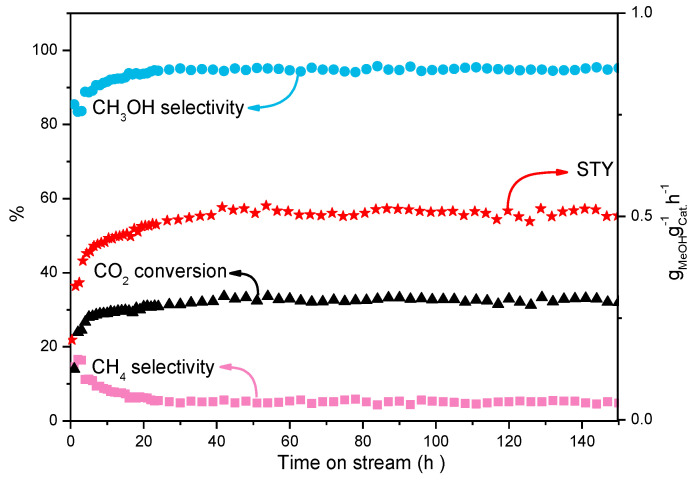
Stability of the MoS_2_-45@C catalyst with granule stacking.

Stability is a fatal issue for the catalysts, which were used in CO_2_ hydrogenation. Under the optimal reaction conditions, the stability of MoCS-45@C was investigated in a fixed-bed reactor, and the results are shown in Figure 6. In the first 20 h, the CO_2_ conversion, the methanol selectivity, and STY were increased with prolonged reaction time, while the methane selectivity was decreased. When the reaction time was more than 20 h, the catalytic performance of MoCS-45@C was kept stable. The CO_2_ conversion was stabilized at around 32.4%, with about 94.8% selectivity of methanol. During the reaction period of 150 h, the catalytic performance of MoCS-45@C showed almost no attenuation, which suggests a promising prospect for industrial applications.

**Figure 7:**
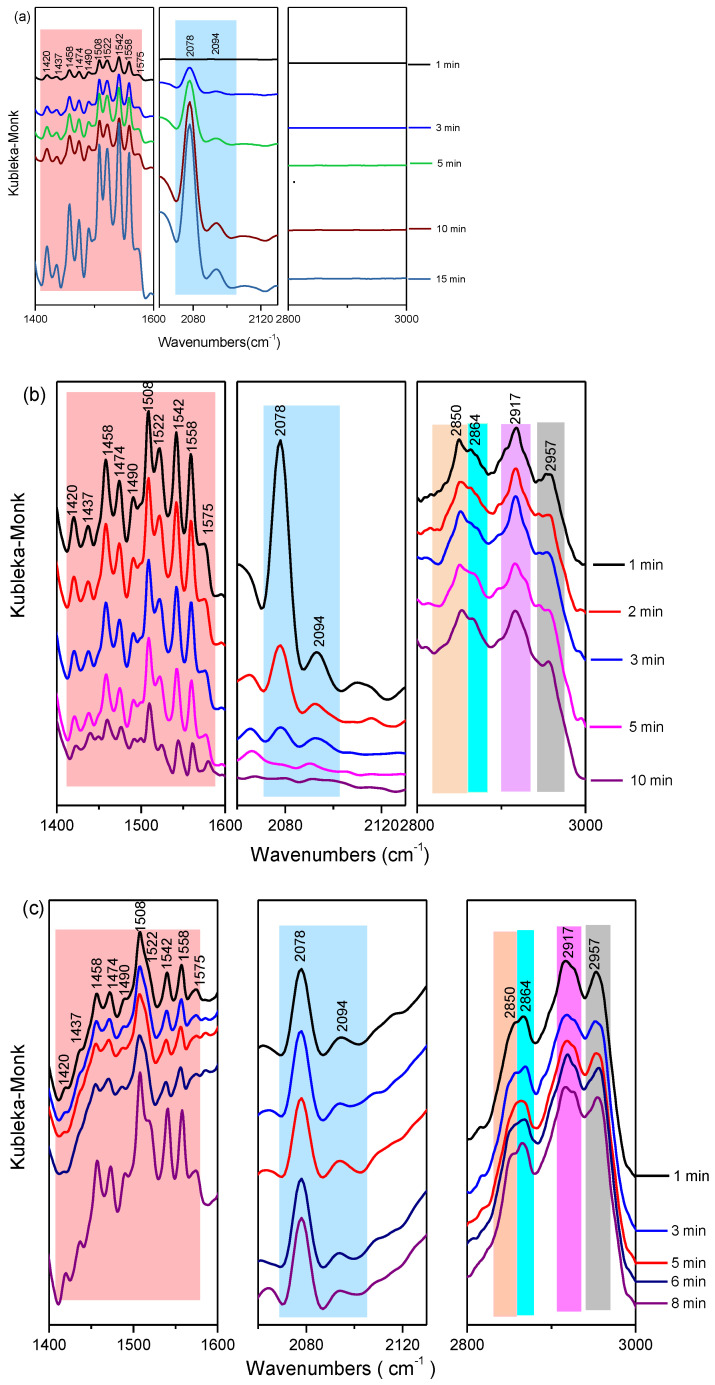
In situ DRIFTS spectra of CO_2_ hydrogeantion on MoS_2_-45@C: different feed introduced to catalyst (**a**) CO_2_; (**b**) H_2_; (**c**) CO_2_ + H_2_.

To propose a reaction sequence and a surface reaction mechanism, in situ diffuse reflectance infrared Fourier transform spectroscopy (in situ DRIFTS) was used to identify the evolution of surface species on the surface of MoS_2_-45@C. The in-situ drift spectra for the hydrogenation of CO_2_ to methanol over MoS_2_-45@C at 180 °C with time were shown in Figure 7, and detailed information on the evolution of intermediate species could be found.

**Figure 8:**
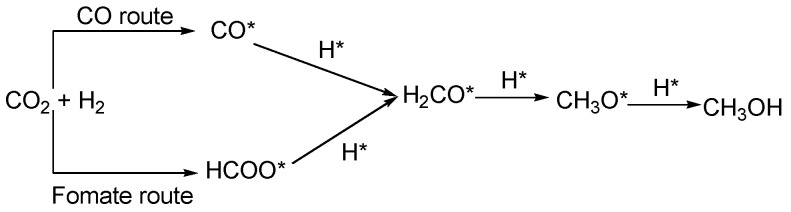
Reaction route for CO_2_ hydrogenation to methanol over MoS_2_-45@C.

Firstly, the pure CO_2_ was introduced into the MoS_2_-45@C catalyst (Figure 7a), and the IR bands at 1420, 1437, 1458, 1522, 1542, and 1575 cm^−1^ were observed, which were assigned to adsorbed *CO_2_ species [59,60,61,62,63]. Moreover, IR bands at 1474 and 1558 cm^−1^ also came into the formation, assigned to bidentate carbonate [59,64], and the signals of monodentate carbonate species were prevalent at 1490 and 1508 cm^−1^ [64,65]. Additionally, their intensities were increased by prolonging the contact time of CO_2_ and MoS_2_-45@C from 1 min to 8 min. These results indicated that CO_2_ could be adsorbed on the surface of the MoS_2_-45@C catalyst, and the adsorption capacity of CO_2_ was improved by prolonging the contact time of CO_2_ and MoS_2_-45@C. It was worth noting that the IR bands at 2078 and 2094 cm^−1^ were found when the MoS_2_-45@C catalyst was exposed to the CO_2_ atmosphere. These results indicated that CO_2_ was dissociated to yield surface-bound CO* on the catalytic surface [56,58,66], and their intensities were increased as time went on. These results would be beneficial for increasing the selective synthesis of methanol from the CO_2_ hydrogenation [31]. Then, the feeding gas was switched from pure CO_2_ to H_2_ (Figure 7b), and the CO* peaks from the dissociation of CO_2_ gradually disappeared with the rise of CH_3_O* peaks (2864 and 2917 cm^−1^) [62,67], and the intensity of which decreased as time went on, thereby indicating the hydrogenation of CO* to CH_3_O* and then the formation of CH_3_OH. At the same time, a weak shoulder peak that appeared at 2957 cm^−1^ in the *ν* (CH) region was also detected, and it was a combination of the CH bending and asymmetric OCO stretching modes of formate species (HCOO*) [60,68]. It was indicated the carbonate species adsorbed on the surface of the MoS_2_-45@C catalyst were also hydrogenated. These results explain the decrease in the peaks intensity of the carbonate species when H_2_ was introduced. Moreover, the IR bonds at 2850 cm^–1^ were also found, and they were assigned to the symmetric and asymmetric H_2_CO* stretching vibrations [60,69], respectively, which might derive from both formate and CO-hydro pathways. These results confirmed that both CO and formate were significant intermediate species for CO_2_ hydrogenation to methanol over the MoS_2_-45@C catalyst. Exposing MoS_2_-45@C to the feed gas (CO_2_ + H_2_), similar in-situ drift IR spectra were obtained (Figure 3c), and these results suggested that the HCOO* hydrogenation route and the CO* hydrogenation route were performed simultaneously in the presence of the MoS_2_-45@C catalyst in the CO_2_ hydrogenation. Hence, combining the results of in-situ DRIFTS with the literature [56,57,58,59,60,61,62,63,64,65,66,67,68,69], we held the opinion that the hydrogenation of HCOO* and CO* were all carried out when MoS_2_-45@C was employed as a catalyst in the CO_2_ hydrogenation (Figure 8).

## 4. Conclusions

In conclusion, comparing MoS_2_/C and bulk MoS_2_ samples, carbon-confining MoS_2_ in MoS_2_-45@C samples prepared by the in-situ pyrolysis method had the characteristics of few layers and small size, which were beneficial to exposing the more active sites. The strong interaction between MoS_2_ and the carbon coating layer in the MoS_2_-45@C catalyst was formed, which was also favorable to the CO_2_ hydrogenation by decreasing the Gibbs free energy of hydrogen adsorption. Moreover, the adsorption capacity of CO_2_ on the MoS_2_-45@C surface was improved when the carbon coating layer was doped with sulfur and nitrogen, which also contributed to the CO_2_ conversion and the methanol selectivity. Under the optimal reaction conditions, the MoS_2_-45@C showed excellent catalytic performance and catalytic stability, and there was no deactivation in CO_2_ hydrogenation for more than 150 h on stream at least, which indicates a promising potential for industrial applications.

## Figures and Tables

**Table 1:** The performance of catalyst in CO_2_ hydrogeantion ^[a]^.

Catalyst	Conversion/%	Selectivity/%			STY
		CH_3_OH	CH_4_	CH_3_OCH_3_	/g_MeOH_ g_cat._^−1^ h^−1^
NSC	-	-	-	-	-
MoS_2_	18.3	66.9	32.7	0.4	0.252
MoS_2_-45@C	27.3	95.8	4.2	0	0.538
MoS_2_-5@C	18.4	79.9	20.1	0	0.302
MoS_2_/C	4.2	78.5	21.5	0	0.068

^[a]^ Reaction conditions: 160 °C, 3 MPa, GHSV 5670 mL g_cat._^−1^ h^−1^, V_H2_/V_CO2_ = 3.

## Data Availability

Not applicable.

## Supplementary Materials

The following supporting information can be downloaded at: https://www.mdpi.com/article/10.3390/ijms23095220/s1 [70,71,72,73,74].
