# Supplementary material for: Exploring the Effects of the Interaction of Carbon and MoS2 Catalyst on CO2 Hydrogenation to Methanol
Source: Int J Mol Sci. 2022 May 7;23(9):5220. doi: 10.3390/ijms23095220 (PMC9104557; doi:10.3390/ijms23095220)
Supplement: Supplementary file 1 [file ijms-23-05220-s001.zip › ijms-1706403-supplementary.pdf]

Exploring the Effects of the Interaction of Carbon and MoS_2 Catalyst on CO_2 Hydrogenation to Methanol

Pingping Cui, Ruyu Sun, Linfei Xiao *, Wei Wu *

College of Chemistry, Chemical Engineering and Materials, Heilongjiang University, Heilongjiang, 150080

Email: xiaolf@hlju.edu.cn; wuwei@hlju.edu.cn

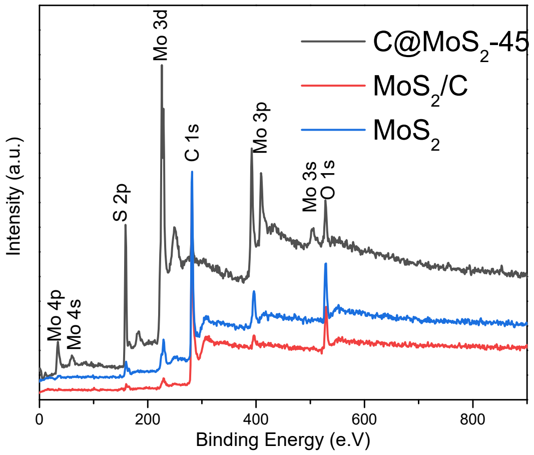

Figure S1. XPS survey spectra

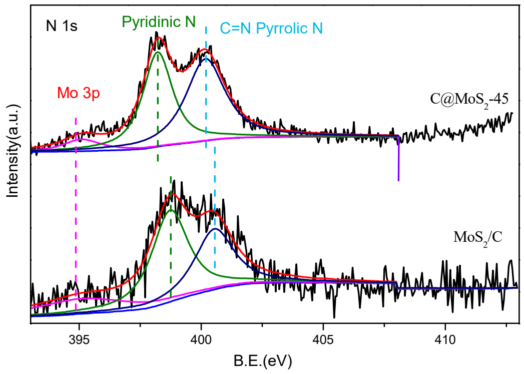

Figure S2. XPS of N1s

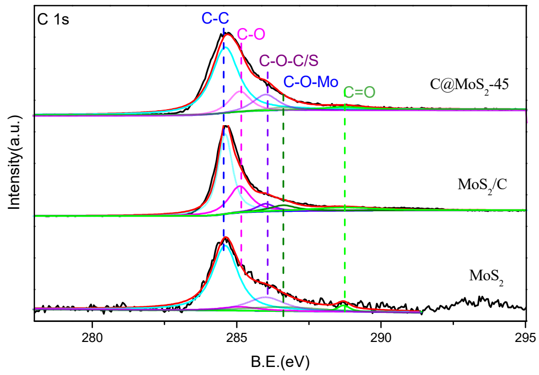

Figure S3. XPS of C1s

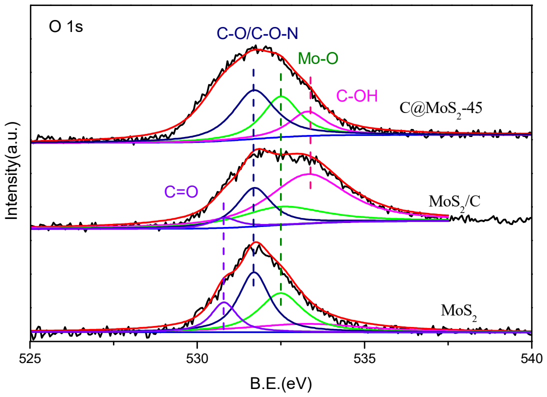

Figure S4. XPS of O1s

Table S1. Mo,S,O,N,C content on the surface of each sample

Catalysts	Mo (mol%)	S (mol%)	C (mol%)	O (mol%)	N (mol%)
C@MoS_2-45	0.58	3.05	73.58	10.52	12.28
MoS_2/C	0.27	0.96	85.38	8.75	4.64
MoS_2	17.31	35.33	28.87	18.4	0.00

Table S2. Qualitative analysis of Mo 3d orbital

Catalysts	Mo^{2+} $3d_{5/2}$	Mo^{2+} $3d_{3/2}$	$Mo^{4+}3d_{5/2}$	$Mo^{4+}3d_{3/2}$	Mo^{6+} $d_{5/2}$	$Mo^{6+}3d_{3/2}$
C@MoS_2-45	228.0 eV	231.1 eV	229.1 eV	232.0 eV	232.4 eV	235.5 eV
MoS_2/C	-	-	229.1 eV	232.0 eV	232.8 eV	235.9 eV
MoS_2	-	-	229.1 eV	232.3 eV	-	-

Table S3. Qualitative analysis of S 2s orbital

Catalysts	Mo-S $S2p_{3/2}$	Mo-S $S2p_{1/2}$	-C-S_x-C- $S2p_{3/2}$	C-S_x-C- $S2p_{1/2}$	C-S(O)$_x$-C- $S2p_{1/2}$
C@MoS_2-45	161.8 eV	163.2 eV	163.8 eV	164.8 eV	168.2 eV
MoS_2/C	161.8 eV	163.2 eV	163.8 eV	164.8 eV	168.2 eV
MoS_2	162.2 eV	163.2 eV	-	-	-

Table S4. Qualitative analysis of N 1s orbital

Catalysts	Mo 3p	Pyridinic N	Pyrrolic N
C@MoS_2-45	395.2 eV	398.2 eV	400.2 eV
MoS_2/C	395.2 eV	398.8 eV	400.5 eV

Table S5. Qualitative analysis of C 1s orbital

Catalysts	$C-C^-$	C-O	C-O-C/S	C-O-Mo	C=O
C@MoS_2-45	284.6	285.2	286.1	286.6	288.7
MoS_2/C	284.6	285.2	286.1	286.6	288.7
MoS_2	284.6	285.2	286.1	286.6	288.7

Table S6. Qualitative analysis of O 1s orbital

Catalysts	C=O	C-O/C-O-N	Mo-O	C-OH
C@MoS_2-45	-	531.7 eV	532.5 eV	533.4 eV
MoS_2/C	530.8 eV	531.7 eV	532.5 eV	533.4 eV
MoS_2	530.8 eV	531.7 eV	532.5 eV	533.4 eV

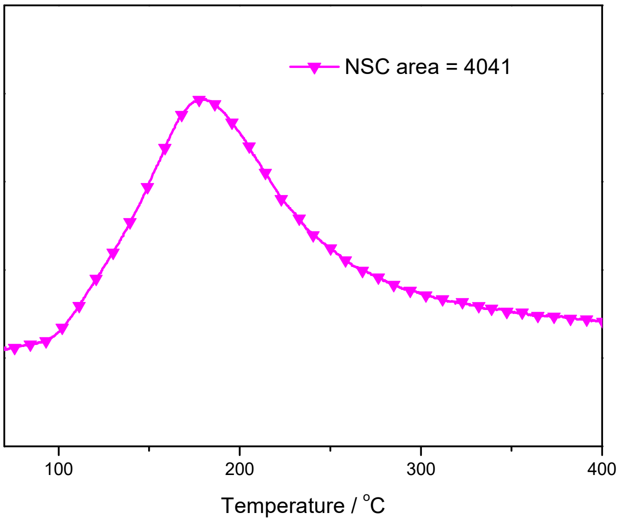

Figure S5. CO_2-TPD curves of NSC

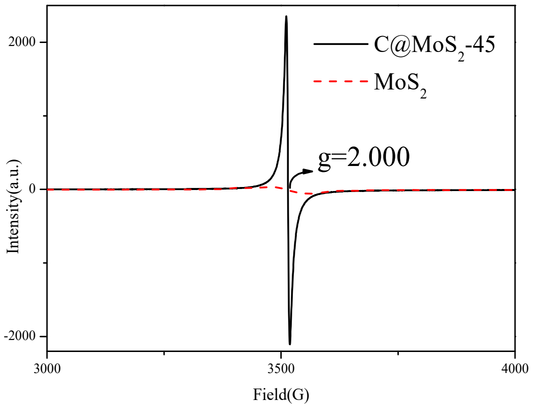

Figure S6. EPR spectrograms of C@MoS_2-45,MoS_2

Electron paramagnetic resonance (EPR) is a magnetic resonance technique originating from the magnetic moments of unpaired electrons and can be used to quantify the amount of unpaired electrons in a sample and to explore the structural properties of its surroundings. [70] Sulphur vacancies can trap unpaired electrons from semi-conducting sulphides and this can be detected by electron paramagnetic resonance (EPR). [71] This determines whether the catalyst contains sulphur vacancies or oxygen vacancies and identifies electrons with residual defects. [72] Quantification of signal intensities from multiple EPR experiments is shown in Figure S6, and based on the fact that EPR intensity is proportional to the concentration of sulphur vacancies, it is clear that the concentration of defect sites is higher in MoS_2-45@C compared to MoS_2. [73,74]
